# Rearing *Trissolcus japonicus* and *Trissolcus mitsukurii* for Biological Control of *Halyomorpha halys*

**DOI:** 10.3390/insects11110787

**Published:** 2020-11-11

**Authors:** Giuseppino Sabbatini-Peverieri, Christine Dieckhoff, Lucrezia Giovannini, Leonardo Marianelli, Pio Federico Roversi, Kim Hoelmer

**Affiliations:** 1 CREA, Research Centre for Plant Protection and Certification, I-50125 Florence, Italy; lucrezia.giovannini@crea.gov.it (L.G.); leonardo.marianelli@crea.gov.it (L.M.); piofederico.roversi@crea.gov.it (P.F.R.); 2 USDA, Agriculture Research Service, Beneficial Insects Introduction Research Unit, Newark, DE 19713, USA; dieck009@umn.edu (C.D.); kim.hoelmer@usda.gov (K.H.)

**Keywords:** brown marmorated stink bug, classical biological control, ovaries, longevity

## Abstract

**Simple Summary:**

*Halyomorpha halys* is a severe agricultural pest of Asian origin, which threatens cultivation of vegetables and fruits worldwide. Classical biological control is foreseen as the most effective approach to reduce populations of *H. halys*. The egg parasitoid *Trissolcus japonicus* is the most important candidate biological control agent. Adventive populations of *T. japonicus* are already present in limited distributions in North America and in Europe. *Trissolcus mitsukurii* is a second Asian species that attacks *H. halys* and has been found in Northern Italy. Efficient laboratory rearing procedures of these biological control agents are needed for release programs to help control the pest. We present data that will help to optimize progeny production with minimal effort.

**Abstract:**

*Halyomorpha halys* is a severe agricultural pest of Asian origin that has invaded many countries throughout the world. Pesticides are currently the favored control methods, but as a consequence of their frequent use, often disrupt Integrated Pest Management. Biological control with egg parasitoids is seen as the most promising control method over the long-term. Knowledge of the reproductive biology under laboratory conditions of the most effective candidates (*Trissolcus japonicus* and *Trissolcus mitsukurii*) for optimizing production for field releases is strongly needed. Rearing of these egg parasitoids was tested by offering three different host supply regimes using new emerged females and aged, host-deprived females in different combinations. Results showed a mean progeny per female ranging from 80 to 85 specimens for *T. japonicus* and from 63 to 83 for *T. mitsukurii*. Sex ratios were strongly female biased in all combinations and emergence rates exceeded 94% overall. Cumulative curves showed that longer parasitization periods beyond 10–14 days (under the adopted rearing regimes) will not lead to a significantly increase in progeny production. However, ageing females accumulate eggs in their ovaries that can be quickly laid if a sufficient number of host eggs are supplied, thus optimizing host resources. Our data showed that offering *H. halys* egg masses to host-deprived female *Trissolcus* once a week for three weeks allowed its eggs to accumulate in the ovary, providing the greatest number of offspring within a three week span.

## 1. Introduction

Over the past three decades the Asian brown marmorated stink bug *Halyomorpha halys* Stål (Hemiptera: Pentatomidae) has become a serious pest worldwide, causing economic damage to a great variety of field crops, vegetables, fruit trees and ornamentals [1]. The disruption of existing integrated pest management programs due to the frequent use of broad spectrum pesticides applied against *H. halys* has caused additional negative effects, including secondary pest outbreaks [2], and the winter aggregations of large numbers of individuals have become a widespread urban nuisance [1,3]. Indirect effects include the transmission of bacteria and yeasts such as *Eremothecium coryli* that may lead to fruit rot [4,5]. Chemical control can be considered as a solution only for the short- and medium-term. The short residual efficacy and the ability of *H. halys* to quickly recover from knockdown, its broad polyphagous behavior and the high mobility of adults across the landscape are the basis for the frequent failure of treatments to provide adequate lasting control [1].

In this scenario, classical biological control should be viewed as an important component of long-term *H. halys* management. In the native range of *H. halys* (northeastern Asia including China, Korea and Japan), the egg parasitoid *Trissolcus japonicus* (Ashmead) (Hymenoptera: Scelionidae) is its most efficient natural enemy and several biological control programs have been planned or are under evaluation around the world (USA, Europe and New Zealand) despite the lack of strict host specificity to *H. halys* by *T. japonicus* [6,7,8,9,10,11,12,13,14,15]. A second egg parasitoid, *Trissolcus mitsukurii* (Ashmead) (Hymenoptera, Scelionidae), has been recorded in Japan to be a common antagonist of *H. halys*, although it has been more closely associated with pentatomids of the genus *Nezara* [16,17]. Thus far, this species has received less attention as a candidate biological control agent of *H. halys.*

Adventive populations of *T. japonicus* were recently discovered in North America and Europe [18,19,20,21] and of *T. mitsukurii* in Italy [19]. Although intentional introductions ex novo of an oligophagous egg parasitoid need to be carefully evaluated within a cost/benefit framework, the rearing and redistribution of specimens of adventive populations of *T. japonicus* in USA and Canada is already underway at various locations, while in Italy a wide scale release plan started in 2020.

Optimal rearing of useful insects in the laboratory needs to balance the need for economic and human resources while maximizing the production of insects [22]. Here we present information to standardize and maximize the laboratory mass-rearing of *Trissolcus* egg parasitoids for field release programs. The use of alternative hosts for rearing rather than *H. halys* has been considered but detailed studies remain to be performed, e.g., assess the adult fitness the preference in the field for the target species instead the host used for laboratory rearing. Despite of the oligophagous host range of both egg parasitoids considered here, their mass production by commercial insectaries is currently only in an embryonic stage as a future prospect. The target host *H. halys* can be reared easily in the lab and egg parasitoids have been reared with success on both fresh and previously frozen egg masses (although a reduction in frozen egg suitability correlated with increased storage time has been reported) [23,24,25]. The use of short-term cold-stored egg masses of *H. halys* was also proposed recently [26].

Biological observations of *T. japonicus* and *T. mitsukurii* under laboratory conditions have been recorded by various authors [16,17,27,28,29]. Both species are able to parasitize an entire *H. halys* egg mass (generally 28 eggs/mass) within few hours of exposure under laboratory conditions [14,28]. Egg mass guarding by parasitizing females is a typical behavior in these egg parasitoids [15,28,30]. The suitability of host eggs is correlated with their age: freshly laid to 3 days old *H. halys* egg masses are more acceptable than older ones [28,29]. Juvenile development is relatively fast, requiring 132.5 degree days with a minimum threshold of 12.2 °C for *T. japonicus* (e.g., 10.5 d at 24 °C) and up to two weeks at 25 °C for *T. mitsukurii*, with males emerging approximately one day before females [6,16,27,28,31]. Adults of both species are long lived, surviving many weeks when fed with honey at 25 °C [17,28].

Female scelionids such as *Gryon* and *Trissolcus* typically mate upon or very soon after emergence with previously emerged males that remained with the egg mass waiting for the emergence of a female [6,28]. Females are able to parasitize host eggs shortly after emerging, but production of offspring is generally higher in the first several days after emergence and it decreases gradually over time and oviposition opportunity [27,28,32]. Considering the costs and benefits from a mass production perspective, the goal is to rear reproductive females for the shortest time with the highest production of progeny possible rather than maintaining them for a longer time with declining fecundity. Balancing the costs of rearing with anticipated output allows researchers to more accurately plan field releases for successful establishment. We present here information for laboratory production of *T. japonicus* and *T. mitsukurii* by comparing the reproductive performance of host-deprived and non-host-deprived females under laboratory conditions.

## 2. Materials and Methods

### 2.1. Origin of Insects

A laboratory colony of *Halyomorpha halys* was established by collecting adults from the field at infested sites in Italy and rearing them in insect cages (BugDorm 4F4545, Insect MegaView Science Co. Ltd., Taichung, Taiwan) in climate-controlled rooms at 26 °C and 16:8 Light/Dark. Adults were fed with plants of *Glycine max* (soybean), fresh vegetables of *Phaseolus vulgaris *(green beans) and *Daucus carota* (carrots), seeds of *Arachis hypogaea* (peanuts) and *Helianthus annuus *(sunflower), fresh fruits of *Malus domestica *(apples), *Actinidia deliciosa *(kiwi) and fresh kernels of *Zea mays* (corn); all sold at the local market. Water was provided with wet cotton. Food was replenished three times per week. Paper towels were hung inside the rearing cage as an oviposition substrate. *Halyomorpha halys* egg masses were collected daily, and 1 day-old egg masses were used in the tests.

Founder material for the laboratory-reared colonies of *T. japonicus* at CREA (Council for Agricultural Research and Economics)-Research Centre for Plant Protection and Certification in Florence (originally collected in Beijing, China) was supplied by the USDA-ARS (United States Department of Agriculture-Agricultural Research Service) Beneficial Insects Introduction Research Unit (BIIRU), in Newark, DE, USA and imported to Italy for laboratory studies under quarantine conditions (authorization number DG/DISR/DISR05/0013647-19/04/2018). *Trissolcus mitsukurii* were field collected at *H. halys*-infested sites in northern Italy [19]. Laboratory reared colonies were established in the rearing facility and specimens used in the tests were obtained from colonies reared over 10 generations. Droplets of pure honey were provided as an adult parasitoid food source and were replaced twice a week. For production of progeny, fresh *H. halys* eggs (1 d-old) were exposed to female *Trissolcus* from the colonies for 24 h. Adults and parasitized host eggs were reared at 26 °C ± 1, 75% RH ± 5 and 16:8 L:D. All stages of *Trissolcus* were reared in glass tubes (15 cm long and 2 cm dia.) closed on both ends with a plastic netting of 250 μm mesh.

Supplemental data on survival and ovarian status of the Beijing population of *T. japonicus* were obtained from experiments conducted previously at BIIRU, Newark, DE, USA. A laboratory culture of *H. halys* initiated from local collections in the Newark area and maintained under the same laboratory conditions and with a variety of foods as described above provided egg masses for maintaining the *T. japonicus* culture and for use in experiments.

### 2.2. Survival Analysis

At BIIRU, fresh female *T. japonicus* were collected from parasitized *H. halys* egg masses within 24 h of emergence. Parasitoids did not have immediate access to food or water upon emergence. Females were then kept individually with a male of approximately the same age for 24 h. For the duration of the experiment, females were held in polystyrene test tubes (12 × 75 mm, 5 mL) and given water and honey if required by the assigned treatment (see below). The tip of a 1.5 mL locking-lid microcentrifuge tube was cut off, a 1 cm long piece of moistened cotton wick inserted, and fastened to the test tube with parafilm. The other end of the tube was covered with a 2 × 2 cm piece of fine cloth mesh and secured over the opening with a snap cap vial with a hole cut into it for the application of honey.

Female parasitoids were randomly assigned to one of the following treatments: (1) no water and no honey (i.e., “starved”); (2) water daily (i.e., “water-only”); (3) water daily and honey on the first day only (i.e., “water + honey-1x”); or (4) water and honey daily (i.e., “water + honey”). All parasitoids were kept in a Percival growth chamber under controlled conditions (21 °C, 40 ± 5% R.H., 16 L:8 D) and water and food were renewed on a daily basis where appropriate. Females were not given opportunities to oviposit in host eggs. Numbers of female *T. japonicus* tested per treatment ranged from 102–109. Individuals that accidentally drowned or were trapped in the honey were included as censored data in the analysis.

### 2.3. Parasitization Tests

At CREA, individual females were randomly selected from the laboratory colony (female proportion of 85% and assumed to be mated), placed in glass tubes as described above, and provided honey drops as a food source. Host egg masses were offered to naïve females for parasitization for 24 h according to the following treatments: (i) 1 d-old females, with host egg masses offered daily for 10 consecutive days (1 d × 10 d); (ii) 7d-old females that were host-deprived until day 7, then successively offered host egg masses daily for 10 consecutive days (7 d × 10 d); (iii) 7 d-old females host-deprived until day 7, then offered host egg masses once a week for three consecutive weeks (w) (7 d × 3 w). To provide host eggs ad libitum, 3 *H. halys* egg masses (22–30 eggs/mass) were attached on card board with wire paper clips. The egg masses were placed in contact with each other to create a single large egg mass in order to avoid female egg mass guarding behavior on a single egg mass without ovipositing in the other two masses. After the 24 h exposure, egg masses were removed from the glass tube, and replenished with a new egg batch when scheduled. Parasitized host eggs were reared in a climatic chamber under the same conditions as described above. Egg masses were checked daily for adult emergence. Tests were replicated 5 times per treatment and species tested. The number of emerged progeny, the sex ratio (N of females/total progeny) and development time (time from host eggs offered to the parent females for parasitization to the emergence of the new F1 adults) were recorded. Unhatched *H. halys* eggs, or those that did not produce an egg parasitoid for two weeks since the last adult emerged were dissected under a stereomicroscope to assess the presence of dead *Trissolcus* pupae or unemerged adults. The difference between the number of emerged egg parasitoids and the dead specimens in the eggs were used to calculate the emergence ratio [28].

### 2.4. Assessment of Ovaries and Egg Load

Egg load over time was assessed for the Beijing *T. japonicus* colony at BIIRU by randomly assigning newly emerged, naïve, mated female parasitoids to one of the following treatments: (1) no water and no honey (i.e., “starved”); (2) water daily (i.e., “water”); (3) water and honey daily (i.e., “fed”). Parasitoids were kept individually in polystyrene test tubes (12 × 75 mm, 5 mL). Treatments (1) and (2) were not continued after 1 d (“starved” and “water”) and 3 d (“water”) because our survival studies showed that survival decreased rapidly in these regimes, with no survival beyond eight days. To monitor egg load development over time in treatment 3, parasitoids were killed in alcohol after 1, 3, 7, 21 and 42 d and stored at minus 7 °C prior to dissection. Egg loads of female parasitoids were assessed by removing the ovaries with the help of fine forceps and insect pins in a droplet of Ringer’s solution under magnification with a dissecting scope. Eggs were stained with a droplet of Neutral Red solution and mature eggs only were then counted.

To assess the total egg load in the ovaries of female *T. japonicus* and *T. mitsukurii* in culture at CREA, newly emerged females were randomly selected from the colonies, kept isolated with no opportunity for oviposition, fed with honey and reared in a climatic chamber under the standard conditions described above. Freshly emerged females (<24 h) and females aged 1, 3, 7, 14, 21 and 28 days were dissected, their ovaries extracted and total egg load was counted after staining eggs with methylene blue [17]. Tests were replicated 10 times for each age and parasitoid species.

### 2.5. Statistical Analysis

Data of progeny production and development time from CREA were tested for normality with the Shapiro–Wilk test and, when necessary, data were square root transformed ($x=\sqrt{x_{i}+1}$); means were separated by ANOVA (Analysis of Variance) and Tukey post-hoc test (*p* < 0.05) [33]. Sex ratio (percentages of females) were arcsine transformed (*x* = $arcsin\sqrt{x_{i}}$) and means were separated by ANOVA and Tukey post-hoc test (*p* < 0.05) (Zar, 2010). Statistical analysis was performed with the software SPSS 20.0 statistical software (IBM Corp., Armonk, NY, USA). Data from the Newark experiments were analyzed with JMP Pro 10.0 for Windows (SAS Institute Inc., Cary, NC, USA). Proportional hazards modeling was applied to assess the effect of treatment and its interaction with parasitoid longevity. Effects of parasitoid treatment, time, and the interaction terms on egg load were analyzed using ANCOVA (Analysis of Covariance).

## 3. Results

### 3.1. Survival Analysis

**Figure 1:**
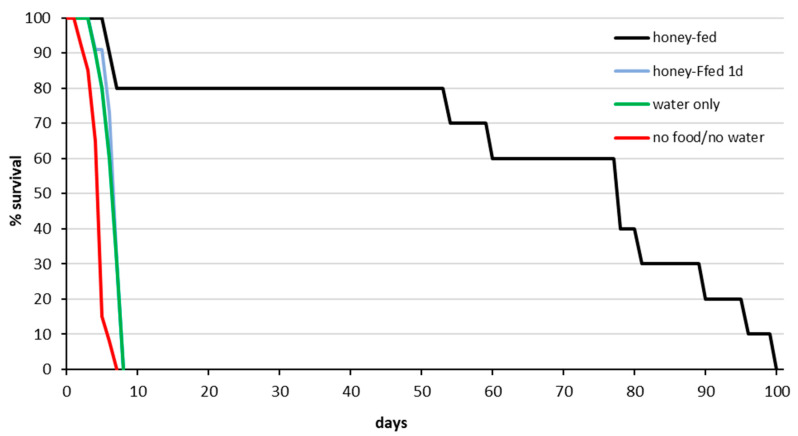
*Trissolcus japonicus* Beneficial Insects Introduction Research Unit (BIIRU) colony: females adult survival rate at different feeding regimes.

Female *T. japonicus* in the Newark BIIRU longevity test lived significantly longer when provided with a regular carbohydrate source, with 80% surviving 55 days and 10% living nearly 100 days. In contrast, females deprived of honey and water from the start, or of honey after the first day, or given only water daily died rapidly after the first several days and none survived longer than 8 days (N = 75, df = 3, Χ^2^ = 45.75, *p* < 0.0001 (Figure 1).

### 3.2. Parasitization Tests

**Figure 2:**
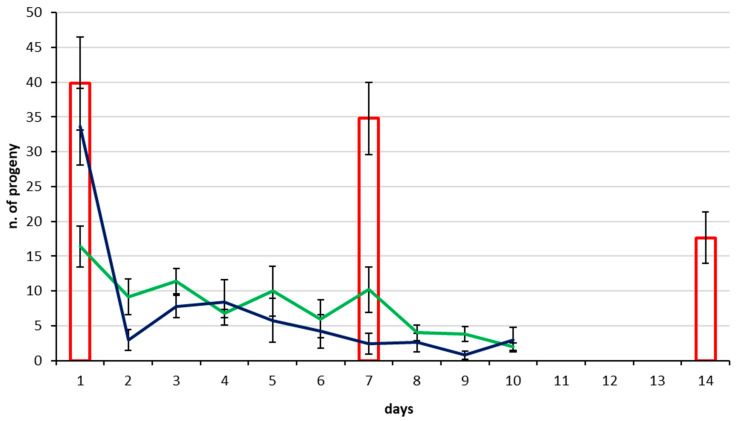
Mean number (±Standard Error S.E.) of progeny produced by *Trissolcus japonicus* Council for Agricultural Research and Economics (CREA) colony under different supply regimes of *Halyomorpha halys* host egg masses. Green line = 1-day-old females with host egg masses offered daily for 10 consecutive days (1 d × 10 d); blue line = 7-day-old females (host deprived until day 7) with host egg masses offered daily for 10 consecutive days (7 d × 10 d); histograms = 7-day-old females (host deprived until day 7) with host egg masses offered once a week for three consecutive weeks (7 d × 3 w); see text for more details.

**Figure 3:**
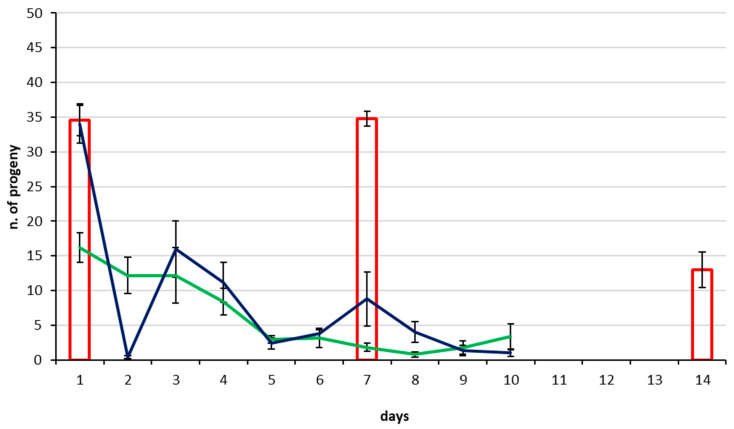
Mean number (±S.E.) of progeny produced by *Trissolcus mitsukurii* CREA colony under different supply regimes of *Halyomorpha halys* host egg masses. Green line = 1-day-old females with host egg masses offered daily for 10 consecutive days (1 d × 10 d); blue line = 7-day-old females (host deprived until day 7) with host egg masses offered daily for 10 consecutive days (7 d × 10 d); histograms = 7-day-old females (host deprived until day 7) with host egg masses offered once a week for three consecutive weeks (7 d × 3 w); see text for more details.

All *T. japonicus* and *T. mitsukurii* females tested by CREA produced progeny starting from their first day of adult life and all produced both male and female progeny indicating they had been mated. No *T. japonicus* died during the two tests with a 10 day host egg supply (1 d × 10 d and 7 d × 10 d), while in the once per week host supply test (7 d × 3 w), two females died in the third week. None of the *T. mitsukurii* females died during the tests. When offering host egg masses daily to 1 d-old females (1 d × 10 d), the highest progeny production occurred on the first day offered, with 16.4 and 16.2 offspring produced, respectively, by *T. japonicus* (Figure 2) and *T. mitsukurii* (Figure 3). Daily production of progeny then declined gradually until the 10th and final day of the experiment, when a mean of two *T. japonicus* offspring and 3.4 *T. mitsukurii* offspring were produced. When females of both *Trissolcus* species were host deprived for seven days (7 d × 10 d and 7 d × 3 w) before first exposure to eggs, the mean number of progeny produced on the first day of egg mass provision was 33.6 for *T. japonicus* and 34.0 for *T. mitsukurii,* respectively. Afterwards, when host egg masses were supplied daily for 10 days (7 d × 10 d), progeny production decreased rapidly on the second day by both egg parasitoid species, following a similar trend over the successive days as the females that had not been host deprived before provision with eggs (1 d × 10 d). By day 10, progeny production declined to a mean of 3.0 for *T. japonicus* and 1.0 for *T. mitsukurii,* respectively. In the third treatment where females were host-deprived for 7 days and thereafter given egg masses once a week for three weeks (7 d × 3 w), mean production of progeny from the first exposure to host eggs resulted in 39.8 offspring for *T. japonicus* and 34.6 for *T. mitsukurii*. After the second week’s exposure to host egg masses a similar high level of progeny was also produced: 34.8 offspring for both species; while after the third week, the mean progeny production decreased significantly to 17.7 for *T. japonicus* and 13.0 for *T. mitsukurii*.

The mean cumulative production of progeny by *T. japonicus* during the different parasitization regimens ranged from 73 to 85, and the sex ratio was strongly female biased in all cases (Table 1). There was no significant difference between progeny production among the three treatments (F_2,12_ = 0.333; *p* = 0.732), but the female sex ratio in the treatment with 7 d-old host-deprived females subsequently given egg masses daily for 10 consecutive days was significantly lower than the other treatments (F_2,12_ = 6.463, *p* = 0.012). Development time within sexes was not significantly different between the three treatments (males: F_2,12_ = 0.455, *p* = 0.645; females: F_2,12_ = 0.297, *p* = 0.749) but was faster in males than females by one day.

The mean cumulative production of progeny by *T. mitsukurii* under the three different parasitization regimens produced a mean offspring ranging from 63 to 83 and sex ratio was strongly females biased in all cases (Table 1). No significant difference was revealed within progeny production among the three treatments (F_2,12_ = 2.584; *p* = 0.117). Sex ratios within each sex were not significantly different among treatments (F_2,12_ = 0.062, *p* = 0.941). Time of juvenile development of males was one day less than for females. It was not significantly different between the three treatments for male progeny (F_2,12_ = 0.549, *p* = 0.591), but was slightly different within treatments for females (F_2,12_ = 11.700, *p* = 0.002).

**Figure 4:**
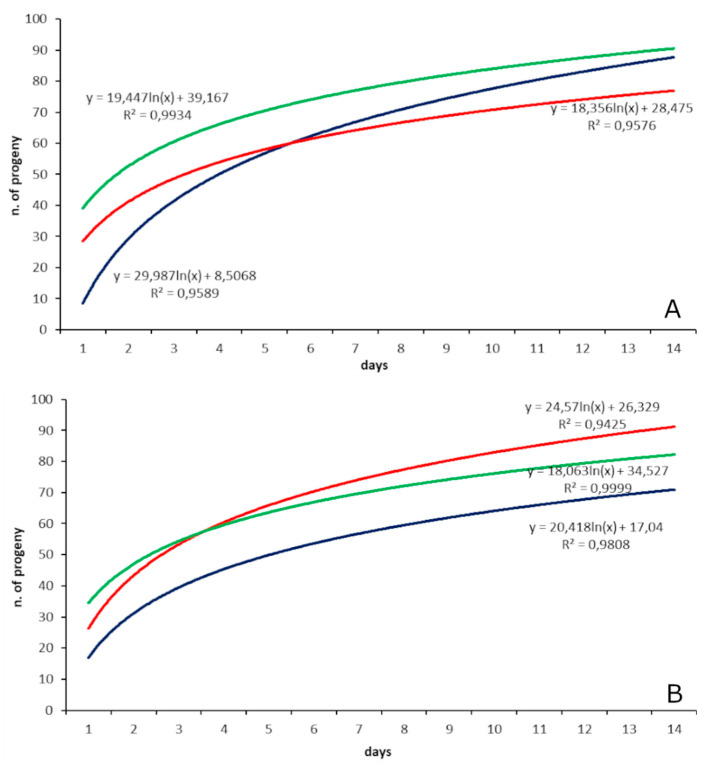
*Trissolcus japonicus* (**A**) and *Trissolcus mitsukurii* (**B**) CREA colonies: regression curves of the cumulative numbers of progeny/female under different supply regimes of *Halyomorpha halys* host egg masses. Green line = 1-day-old females with host egg masses offered daily for 10 consecutive days (1 d × 10 d); blue line = 7-day-old females (host deprived until day 7) with host egg masses offered daily for 10 consecutive days (7 d × 10 d); red line = 7-day-old females (host deprived until day 7) with host egg masses offered once a week for three consecutive weeks (7 d × 3 w); see text for more details.

The regression analysis of the cumulative progeny production by *T. japonicus* and *T. mitsukurii* between the three different treatments of parental females resulted in saturation-like curves, and the corresponding equations are shown on the graphs (Figure 4). The daily increment of additional progeny production by *T. japonicus* one-day-old females given host eggs for 10 days and 7 d-old host-deprived females subsequently offered eggs for 10 consecutive days (1 d × 10 d and 7 d × 10 d) after day 4 and day 3 of exposure, respectively, was less than 10% of the total progeny produced during the first 2 to 3 days; by day 9, the added daily progeny production contributed only 4.07% and 2.73%, respectively, for the two treatments. For 7 d-old host-deprived females with host egg masses thereafter offered once a week for three consecutive weeks (7 d × 3 w), the increment of additional progeny during the second week was already less than 15% of the total progeny produced. Similar trends were observed for *T. mitsukurii*: for 1d-old females and 7 d-old host-deprived females given eggs for 10 consecutive days (1 d × 10 d and 7 d × 10 d) the daily increment of added progeny production declined to less than 10% starting from the 3rd day of eggs given in both treatments; and from day 9, the added daily progeny production contributed only approximately 3% of the total. For the 7 d-old host-deprived females given egg masses once a week for three consecutive weeks (7 d × 3 w), the added production of progeny during the second week was approximately only 15% of the total treatment production.

### 3.3. Egg Load in the Ovary

**Figure 5:**
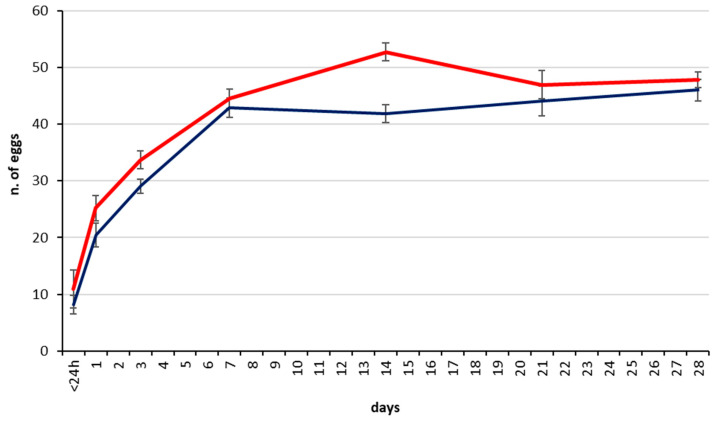
*Trissolcus japonicus* (blue line) and *Trissolcus mitsukurii* (red line) CREA colonies: total number of mature eggs in the ovary at different time intervals in females with no oviposition opportunity (bars indicate S.E.).

**Figure 6:**
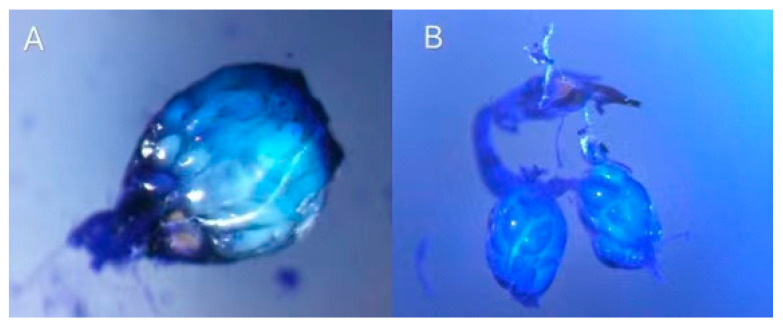
*Trissolcus mitsukurii:* full egg load in the abdomen (urotergum removed from abdomen) (**A**) and dissected reproductive organs showing ovaries, oviduct and ovipositor (**B**).

The mean number of mature eggs in the dissected ovaries of newly hatched females (<24 h) assessed in the CREA Florence experiments was 8.2 ± 1.63 S.E. for *T. japonicus* and 10.9 eggs ± 3.37 S.E. for *T. mitsukurii* (Figure 5). One day after emergence this number had increased to 20.04 ± 2.11 S.E. for *T. japonicus* and 25.20 ± 2.24 S.E. for *T. mitsukurii*, and by day 3, the mean egg load of *T. japonicus* was 29.1 (±1.25 S.E.) and 33.70 (±1.59 S.E) for *T. mitsukurii*. By day 7 and through the end of the experiment on day 28 the mean egg load in the ovary was consistently between 41.9 ± 1.58 S.E. and 46.0 ± 1.87 S.E. in *T. japonicus* and between 44.5 ± 1.68 S.E. and 52.7 ± 1.57 S.E. eggs in *T. mitsukurii* (Figure 6).

**Figure 7:**
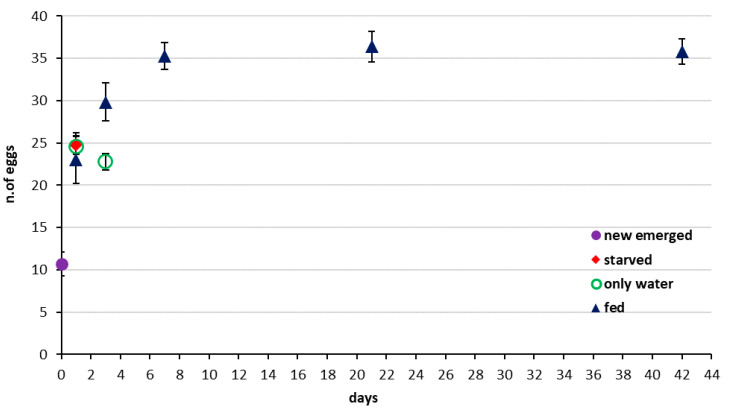
*Trissolcus japonicus* BIIRU colony: total number of mature eggs in the ovary at different time intervals in females with no oviposition opportunity (bars indicate S.E.).

The CREA assessment of *T. japonicus* ovarian egg load was comparable with the initial laboratory assessment of the same population at Newark BIIRU several years earlier (Figure 7). In that study, ovaries held a mean of 10.6 ± 1.9 S.E. upon emergence at day 0. Females regularly provided with honey had a mean egg count of 22.9 ± 5.9 S.E. one day later, 29.65 ± 4.5 S.E. by day 3, and from 35.3 ± 3.2 S.E. to 36.5 ± 3.7 S.E. from day 7 through 42. Females given no water or honey had slightly higher means of 24.15 ± 2.2 S.E. on day 1, and females given only water subsequently had a significantly reduced egg load by day 3 of 22.35 ± 1.9 S.E.

## 4. Discussion

In order to develop a practical and successful applied biological control program, it is important to optimize laboratory rearing procedures so that production of biocontrol agents is maximized while also using labor and material resources efficiently. The urgent need for a biological control option for managing *H. halys* in many areas of the world points to the need for an efficient rearing method of its biological control agent. Mass-rearing the host to support rearing large numbers of its natural enemy is often expensive and time consuming, and optimal use of this resource is a crucial aspect in such rearing programs [23,24,34].

Our results show that, as seen in other scelionids (e.g., *Gryon*), the capacity for oviposition by *Trissolcus* is highest during the first several days following emergence of a newly reproductive female. More than 90% of the progeny of *Gryon pennsylvanicum* (Ashmead) (Hymenoptera: Scelionidae) were produced within the first two weeks of its emergence [32]. With *T. japonicus* and *T. mitsukurii* we observed similar trends, confirming observations of *T. japonicus* by [28] who reported that progeny production peaked on the second day of adult female life. Our results showed that more than 78% of *T. japonicus* eggs were laid within the first 5 days of oviposition during a 10 to 21 day experiment with different host exposure treatments. This trend was also observed with *T. mitsukurii* when parasitizing eggs of *Nezara viridula* L. (Hemiptera: Pentatomidae), with offspring production decreasing abruptly after the second day of exposure to host eggs so that only a few host eggs were subsequently parasitized [27]. Given the fact that *H. halys* egg masses are generally composed of 28 eggs, one egg mass is more than sufficient to fully exploit the daily parasitization capacity of freshly emerged females. However, unparasitized host eggs represent potential waste of resources in a rearing program since neither *T. japonicus* nor *T. mitsukurii* exceeded production of two dozen offspring within a 24 h time span. Scelionids are known to be able to accumulate eggs in their ovaries when facing host deprivation, with their parasitization capacity increasing over time as a result [32]. The accumulation of eggs in the ovary of *T. mitsukurii* was described by [17], who showed that females doubled their supply of eggs in ovaries within a week of adult life (although the number of eggs reported was much higher than those we observed in the present study). Our results showed that female *T. japonicus* and *T. mitsukurii* that were host-deprived for 7 days were able to accumulate their eggs and then deposit them all within a 24 h oviposition opportunity, in some cases with over 4 dozen offspring produced in one day of parasitization. When host eggs were again offered on a daily basis, progeny production subsequently decreased rapidly to levels observed in the experimental treatments with females that were not host-deprived. When host egg masses were supplied once a week in successive weeks to the parental females, allowing a gradual re-accumulation of eggs in the ovary for a second and third week, progeny production remained high (especially in the second week). This approach permitted us to obtain a high level of *Trissolcus* production for at least three weeks and to concentrate the production within a few days of emergence of each generation. In turn, this allowed us to more efficiently operate our rearing facilities and optimize resources. However, progeny production over time appeared quite similar in all three rearing conditions. Thus, with a daily host egg supply, the number of eggs offered (entire egg masses or smaller clusters of them) must be adjusted to minimize the occurrence of unparasitized host eggs which then hatch. Longevity data of female *T. japonicus* from the Newark BIIRU colony indicated a potentially long lifespan of two to three months, but these results were obtained with females that were not given opportunities to oviposit. [17,28] reported notably shorter lifespans for *T. japonicus* and *T. mitsukurii*, respectively, as well as a progressive decline in progeny production in older females. Ongoing research with *T. japonicus* (unpublished Newark BIIRU data) indicates that frequent oviposition entails a cost of reduced lifespan, and that sperm depletion gradually leads to a decreasing female:male ratio. Further studies with re-mated females are, therefore, needed to better define the upper limits of productivity. However, based on our studies, a rearing protocol based upon two to three weeks of female exposure to host eggs offers a reasonable framework for mass production of *Trissolcus* in the laboratory. Taking advantage of their capacity to successfully attack several entire *H. halys* egg masses within a single day, more than one egg mass may be offered as long as they are set close to each other so that ovipositing females respond to the eggs as if belonging to a single large egg mass.

Analysis of *T. japonicus* and *T. mitsukurii* egg loads by dissection of their ovaries explains the ability of females to produce the large numbers of progeny observed in our oviposition tests. Maximum daily production ranged from forty to fifty progeny by 7-day-old host-deprived females, which corresponds with the maximum egg load of 43 in the ovary of 7d-old *T. japonicus* and 45 in *T. mitsukurii*. The full egg load in the ovary may be determined by the available space in the abdomen, since in all other successive ages of females examined the egg load did not change significantly in either parasitoid species.

The development time of *T. japonicus* and *T. mitsukurii* from oviposition to adult emergence observed in our study was consistent with other reported studies: approximately 11–12 days [27,28,31] although our results were one to two days less than what was observed by [16] for *T. mitsukurii*.

The emergence rate was nearly 95% or greater in all treatments for both parasitoids. This was higher than reported by [17] for *T. mitsukurii* at similar temperatures, and similar or higher than the ones reported by [29,31] and [13] for *T. japonicus* under similar rearing conditions.

The female sex ratio was also high in all treatments tested, mostly between 80 and 90% and consistent with rates similar to those reported in other studies of both parasitoids [10,25,27,28,31]. The sex ratio in *T. japonicus* varied among treatments in the present work but was never less than the ratios reported by [8] for similar rearing conditions.

## 5. Conclusions

Development of optimal rearing methods for *Trissolcus* species, with special attention to *T. japonicus*, is a crucial step for eventual control of *H. halys* populations with biological control programs. Accurate data on *Trissolcus* biology and reproduction under laboratory conditions is needed to accurately plan for optimal rearing procedures, thus obtaining this information is one of the key steps that will enable the success of field releases. Our data showed that offering *H. halys* egg masses to host-deprived female *Trissolcus* once a week for three weeks allowed its eggs to accumulate in the ovary, which in turn resulted in the production of the greatest number of offspring within a three week span. Dissections of female wasps confirmed that the maximal egg load in ovaries was reached at about a week after female emergence, and remained more or less constant at approximately 45–55 eggs. Females held in a host-deprived condition for shorter periods can also be used for rearing, but we suggest maintaining a minimum of 4–5 days between each interval of host provisioning to permit optimal accumulation of eggs in the ovary, as was also reported by [13]. Conversely, supplying host egg masses to newly emerged females on a daily basis will instead result in a production of approximately a dozen offspring per day/female initially, but this level will decrease over time. Progeny production, sex ratio and development time were not significantly different among the three treatments tested, indicating that the three tested regimes are essentially equivalent for minimizing the inefficiency of leaving host eggs unparasitized. The similarity of response by these two egg parasitoids suggests that our rearing protocols could be useful starting points for other *Trissolcus* species used in other biological control programs.

## Figures and Tables

**Table 1:** *Trissolcus japonicus* and *Trissolcus mitsukurii*: number of progeny produced, sex ratio and development time using *Halyomorpha halys* eggs as host and under the following test conditions: 1 d × 10 d = 1-day-old females offered new host egg masses daily for 10 consecutive days; 7 d × 10 d = 7-day-old females (host deprived until day 7) offered new host egg masses daily for 10 consecutive days; 7 d × 3 w = 7-day-old females (host deprived) offered host egg masses once a week for three consecutive weeks. Values followed by different letters within columns are significantly different (*p* < 0.05); brackets indicate min. and max. range. Significant differences among the three treatments were assessed separately for each species.

Species	Treatment	n. Progeny/Female	Sex Ratio (% of Females)	Develop. Time Males (d)	Develop. Time Females (d)	Emergence Rate (%)
*Trissolcus japonicus*	1 d × 10 d	79.8 (63–88) a	84.6(71.3–90.9) a	11.3 (9–15) a	12.2 (11–15) a	98.5
	7 d × 10 d	73.4 (40–121) a	78.3 (71.9–85.5) b	11.2 (10–14) a	12.2 (11–14) a	98.9
	7 d × 3 w	85.2 (46–121) a	91.4 (88.4–95.4) a	11.1 (10–13) a	12.0 (11–15) a	99.63
*Trissolcus mitsukurii*	1 d × 10 d	63.0 (52–75) a	88.1 (75.9–94.3) a	11.3 (11–13) a	12.3 (11–14) a	94.60
	7 d × 10 d	83.0 (56–103) a	89.8 (85.7–91.3) a	11.5 (11–13) a	12.5 (11–15) a	95.42
	7 d × 3 w	77.2 (66–90) a	88.5 (84.4–94.6) a	11.5 (10–13) a	12.0 (12–13) b	96.63
